# MicroRNAs as Biomarkers in Amyotrophic Lateral Sclerosis

**DOI:** 10.3390/cells7110219

**Published:** 2018-11-20

**Authors:** Claudia Ricci, Carlotta Marzocchi, Stefania Battistini

**Affiliations:** Department of Medical, Surgical and Neurological Sciences, University of Siena, 53100 Siena, Italy; carlottamarzocchi@libero.it (C.M.), stefania.battistini@unisi.it (S.B.)

**Keywords:** amyotrophic lateral sclerosis (ALS), biomarker, microRNA, cerebrospinal fluid (CSF), muscle biopsy, circulating miRNAs

## Abstract

Amyotrophic lateral sclerosis (ALS) is an incurable and fatal disorder characterized by the progressive loss of motor neurons in the cerebral cortex, brain stem, and spinal cord. Sporadic ALS form accounts for the majority of patients, but in 1–13.5% of cases the disease is inherited. The diagnosis of ALS is mainly based on clinical assessment and electrophysiological examinations with a history of symptom progression and is then made with a significant delay from symptom onset. Thus, the identification of biomarkers specific for ALS could be of a fundamental importance in the clinical practice. An ideal biomarker should display high specificity and sensitivity for discriminating ALS from control subjects and from ALS-mimics and other neurological diseases, and should then monitor disease progression within individual patients. microRNAs (miRNAs) are considered promising biomarkers for neurodegenerative diseases, since they are remarkably stable in human body fluids and can reflect physiological and pathological processes relevant for ALS. Here, we review the state of the art of miRNA biomarker identification for ALS in cerebrospinal fluid (CSF), blood and muscle tissue; we discuss advantages and disadvantages of different approaches, and underline the limits but also the great potential of this research for future practical applications.

## 1. Introduction

Amyotrophic lateral sclerosis (ALS), the most common adult-onset neurodegenerative disorder, is an incurable and invariably fatal condition characterized by the progressive loss of motor neurons in the motor cortex, brain stem, and spinal cord [1]. Motor neurons are selectively affected by degeneration and death, however the collective evidence is that ALS is non-cell autonomous, but rather pathogenesis and disease progression depend on the active participation of non-neuronal neighboring cells such as microglia, astrocytes, muscle and T cells [2,3]. Motor neuron degeneration causes progressive weakness of limb, thoracic, abdominal, and bulbar muscles.

During the early stages of the disease symptoms may vary depending on dysfunction of upper motor neurons (UMN) in the motor cortex (resulting in hyperreflexia, extensor plantar response, and increased muscle tone), or lower motor neuron (LMN) in the brainstem and spinal cord (leading to generalized weakness, muscle atrophy, hyporeflexia, fasciculations, and muscle cramps) [1]. Patients with bulbar onset ALS usually develop slurred and nasal speech and difficulty chewing or swallowing. Bulbar onset occurs less frequently than limb involvement, and accounts for about 25% of ALS cases. During the disease course, most cases show the presence of both LMN and UMN signs affecting spinal and brainstem regions [4]. Death, mainly due to bulbar dysfunction and respiratory insufficiency, occurs within 2–4 years of first symptoms; however, a small group of patients with ALS may survive for 10 or more years [5].

### 1.1. Epidemiology and Genetic Factors

The incidence of ALS is 2.1 per 100,000 persons per year, with an estimated prevalence of 5.4 cases per 100,000 population [6]. Based on data collected by population-based registers, the incidence of ALS increases after the age of 40, shows a peak in the late 60s or early 70s, and then displays a fast decline [7]. The reported male to female ratio varies widely with the age: a sex ratio of 2 or higher is observed for younger patients, while it appears to decrease towards 1 when the proportion of older patients increases [8]. Over the years, several environmental and lifestyle risk factors have been suggested as potential contributors to the cause of ALS. Nevertheless, no conclusive data are yet available, and further studies are required to identify exogenous risk factors of ALS [7,9].

Most cases (around 90%) are classified as sporadic ALS (SALS), since they are not associated with a documented family history. In 1–13% of patients the disease is inherited and defined as familial ALS (FALS), most frequently with a Mendelian dominant inheritance and high penetrance, even though pedigrees with recessive inheritance or incomplete penetrance have been described [10]. The mean age of onset for FALS is 46 years and for SALS is 56 years. In familial ALS, age of onset displays a Gaussian distribution, whereas an age-dependent incidence characterizes sporadic ALS [4]. Disease with an onset prior to 25 years of age is defined as “juvenile ALS” [11]. Apart from the mean age of onset, sporadic and familial forms are clinically indistinguishable suggesting a common pathogenesis.

Several genes have been associated with pathogenesis of ALS. The most common ALS causative genes include chromosome 9 open reading frame 72 (*C9orf72*), Cu2+/Zn2+ superoxide dismutase (*SOD1*), TAR DNA-binding protein 43 (*TARDBP*), and RNA binding protein FUS (*FUS*) [12,13,14], but a lot of other genes have been associated with the disease [15]. Notably, the mutated genes in ALS encode for proteins with very distinct functions in the cell. However, interestingly many ALS-linked genes, particularly *TARDBP* and *FUS*, are involved in RNA metabolism, including microRNA (miRNA) processing [16,17].

### 1.2. Diagnosis and Treatment

There is no objective laboratory test able to provide the diagnosis of ALS, which remains mainly based on clinical assessment, electrophysiological examinations, and exclusion of conditions that can mimic ALS. The certainty level of the diagnosis of ALS may be classified into different categories by clinical and laboratory assessments based on El Escorial criteria [18].

Currently, riluzole and edaravone represent the only drugs approved by the FDA for ALS, providing however a limited improvement in survival [5]. The most significant benefit of riluzole is observed after intervention in the early stages of the disease [19]. Thus, an early diagnosis of ALS could provide the most effective results. Since diagnosis of ALS relies on clinical symptoms, and the time from the first symptoms to diagnosis is about 12 months, there is a delay hindering a successful therapy [5]. This phenomenon underlies the importance of the development of screening tests able to detect the disease in early stages.

## 2. Role of Biomarkers in ALS

In the last years, research has been focused on the identification of potential biological markers to use in diagnostic procedure and clinical practice.

According to the National Institutes of Health Biomarkers Definitions Working Group, a biomarker is defined as “a characteristic that is objectively measured and evaluated as an indicator of normal biological processes, pathogenic processes, or pharmacologic responses to a therapeutic intervention” [20]. Biomarkers can be classified into three general categories: (1) diagnostic biomarkers, which are used for differential diagnosis; (2) prognostic biomarkers, which can differentiate a good or a bad outcome of the disease; and (3) predictive biomarkers, which are utilized for assessing whether a treatment may be effective for a specific patient or not.

In the case of ALS, biomarkers would allow an earlier and more accurate diagnosis, with the opportunity to start an earlier treatment able to modify the disease course. They could help the classification/stratification of ALS patients, monitor the disease progression and identify patients who will respond better to a particular drug. Biomarkers can also provide a valuable tool for the identification of new therapeutic approaches and drive patients’ enrollment in clinical trials. Furthermore, they may represent a link between the results obtained in animal models and the human patients, providing insight on potential therapeutic targets. 

Over the last two decades, intensive work has been carried out to find consistent biomarkers for ALS. Several candidates involved in excitotoxicity, oxidative stress, neuroinflammation, metabolic dysfunction, and neurodegeneration processes have been explored [21], but, unfortunately, none of these biomarkers has been currently translated into a practical diagnostic tool.

## 3. miRNAs as Biomarkers

Recently, among the different categories of potential biomarkers, miRNAs have aroused great interest in several fields of research. miRNAs are short (about 22 nucleotides in length) non-coding RNA molecules that play an important role as endogenous regulators of gene expression acting at the post-transcriptional level. miRNAs are synthesized from primary miRNAs, which are transcribed in the nucleus. Primary miRNAs are processed into pre-miRNAs by Drosha and then exported to the cytoplasm. Pre-miRNAs are eventually processed by the Dicer complex, resulting in mature miRNAs, which form RNA-induced silencing complexes [22]. miRNAs have a tissue-specific expression and this knowledge can help to better understand a normal and a disease development of the respective tissue [23]. miRNAs are known to play important roles in many physiological and pathological processes, including tumorigenesis [24], metabolism [25], immune function [26], and several neurodegenerative disorders [27], such as Parkinson’s disease, Alzheimer’s disease, Huntington’s disease [28] and also ALS [29].

miRNAs have several intrinsic characteristics that make them promising as biomarkers. An ideal biomarker should display high sensitivity, specificity, and predictive power. miRNAs have been shown to have high specificity, and, in particular in cancer research, where a plethora of publications has been generated, it has been demonstrated that miRNA expression profiles differ among cancer types according to diagnosis and developmental stage of the tumor, with a better resolution than traditional gene expression analysis [30]. Moreover, unlike other RNA classes, miRNAs are remarkably stable and therefore can be robustly measured in many biological body fluids including plasma, tears, saliva and cerebrospinal fluid [31]. Indeed, miRNAs appear resistant to boiling, repeated freeze-thawing cycles, pH changes, and fragmentation by chemical or enzymes [32,33,34]. Furthermore, recent evidence indicates that miRNAs can be detected in biological fluids and can be used to “capture” changes in the cells of origin, including neurons [35].

In addition to these general considerations, several findings suggest a specific involvement of miRNAs in ALS. For example, the loss of Dicer is sufficient to cause progressive degeneration of spinal motor neurons [36]; in addition, a global down-regulation of miRNAs is a frequent molecular denominator for multiple forms of human ALS [37]. Moreover, a common theme for several ALS-related genes is a role in RNA processing pathways [38]. FUS facilitates co-transcriptional Drosha recruitment to specific miRNA loci [39] and TARDBP participate to miRNA biogenesis as a component of both Drosha and Dicer complexes [16].

### miRNA Detection

During the last decade, the development of methods for detecting miRNAs has risen to become a very attractive area of research. Although miRNAs have characteristics that made them suitable biomarkers, the detection of these molecules is challenging due to their intrinsic characteristics including small size, sequence similarity among various members, low level and tissue-specific or developmental stage-specific expression. Two approaches commonly used in the research of miRNAs as biomarkers, including studies in the area of neurodegenerative diseases and in particular in ALS, are reported below.

(1) Measurement of hundreds of miRNAs in specimens from patients with a pathology of interest and from control subjects using profiling methods, such as microarray, quantitative Real-Time Polymerase Chain Reaction (qRT-PCR)-based array, quantitative nCounter or Next Generation Sequencing (NGS), with subsequent validation of identified miRNAs by qRT-PCR;

(2) Analysis of selected miRNA(s) already known as related to specific tissues, cell types, or gene expression pathways. In this case, the number of miRNA(s) to be tested is limited, which makes the use of individual qRT-PCR appropriate, increasing sensitivity and reproducibility of the analysis.

Among the profiling methods, microarray is a powerful high-throughput widely used tool that screens large numbers of miRNAs analyzing simultaneously several samples processed in parallel in a single experiment [40]. An alternative method is deep-sequencing, which relays on NGS machines that can process millions of sequence reads in parallel in just a few days [41,42]. Sequence reads are processed by bioinformatics analysis, which identifies both known and novel miRNAs in the data sets, and perform a relative quantification using a digital approach [43]. Finally, qRT-PCR arrays can also be used to detect multiple miRNAs at the same time [44]. This approach is able to detect miRNAs in very low copy number [45]. This is an important aspect, since large amounts of RNA from clinical samples can be difficult to obtain. Other advantages of qRT-PCR-based techniques used in routine diagnostic are sensitivity, specificity, speed and simplicity [46]. Of note, potential biomarkers selected by array-based analysis need to be confirmed by qRT-PCR, due to high variability and low reproducibility of results obtained from these techniques [47].

A critical issue in qRT-PCR analysis is the data normalization approach. Normalization refers to adjusting for variations in data that are due to known factors (usually technical factors) and not related to the biological differences that are being investigated, and that could otherwise lead to inaccurate quantification. For this reason, stable normalizers are needed, but identifying such molecules is challenging, and it is often necessary to select them on a case-by-case basis [48]. Normalization to reference invariant miRNAs [49] is effective in many cases, but this approach requires that the reference miRNA is not influenced by the condition being studied. Exogenous spike-in controls added to samples during the miRNAs extraction may be used to compensate the variability caused by extraction efficiency and possible presence of inhibitors [50]. The combined use of two or more normalizers usually allows reducing experimental variability and improving reliability of the analysis.

## 4. miRNAs as Biomarkers for ALS

**Figure 1:**
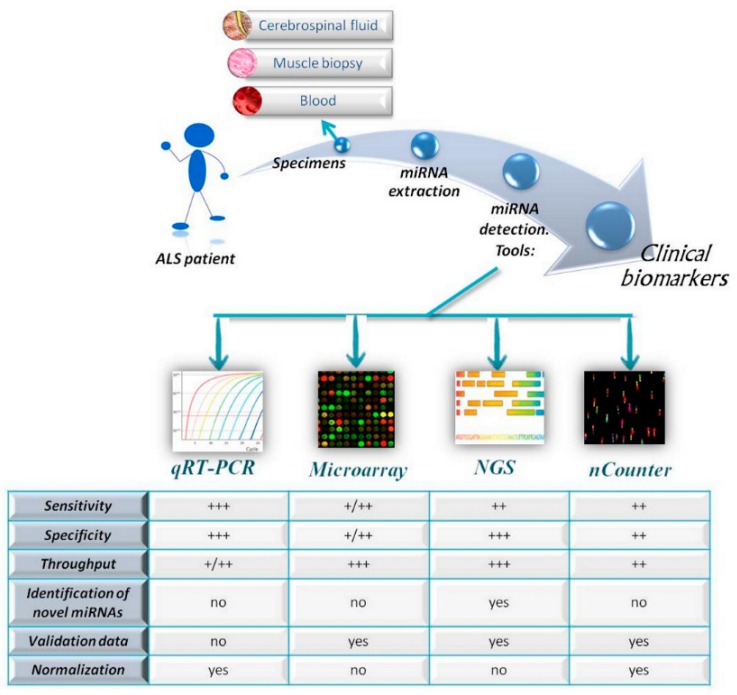
MicroRNA (miRNA)-based biomarkers in amyotrophic lateral sclerosis (ALS) patients. Schematic workflow to identify possible miRNAs as biomarkers starting from ALS patients’ sample using different quantitative approaches. The comparison among the common characteristics of miRNA detection platforms is summarized in the figure. Sensibility, specificity and throughput are classified as follows: +++ (very high), ++ (moderate), +/++ (moderate to low) and + (low). Abbrevations: qRT-PCR, quantitative Real-Time Polymerase Chain Reaction; NGS, Next Generation Sequencing.

The first paper about miRNAs as biomarkers for ALS in human samples was published by De Felice and colleagues in 2012 [51]. Since then, a large number of studies have been performed on cerebrospinal fluid (CSF), blood and muscle biopsies from ALS patients. See Figure 1 for a schematic workflow of identification of miRNA-based biomarkers.

### 4.1. miRNAs in Cerebrospinal Fluid 

Cerebrospinal fluid (CSF) is the fluid that bathes the central nervous system (CNS) and, due to this direct interaction, represents a potentially ideal source for identifying biomarkers for ALS. miRNAs present in CSF can mirror CNS physiological and pathological conditions representing more sensitive biomarkers of brain changes than those present in other biofluids [35]. The presence of miRNAs in CSF was first demonstrated by Cogswell and colleagues [52]. The authors reported that the amount of miRNAs secreted or excreted from other organs to CSF is very limited and that the major source of miRNAs detected in CSF are immune cells present in this biofluid. In addition, other studies showed that the miRNAs present in CSF derived also from neurons [53].

CSF samples are obtained by lumbar puncture, a procedure used for diagnostic purposes to confirm ALS diagnosis and exclude other pathologies, as inflammatory nerve conditions. Lumbar puncture, however, represents an invasive procedure, that cannot be repeated during the disease course for ethical implications. Thus, analysis of miRNAs in CSF is not suitable to identify biomarkers to follow disease progression.

Up to date, five studies have been published about the identification of miRNAs as biomarkers in CSF from ALS patients. Results are shown in Table 1. 

The first three studies in Table 1 selected a limited set of miRNAs to analyze: 43 miRNAs found up-regulated in SOD1 spinal cord CD39+ microglia and splenic Ly6Chi monocytes [54], a group of *TARDBP* binding miRNAs [55], or one selected miRNA, over-expressed in ALS blood leucocytes [56], respectively. The other two studies performed a miRNA expression profiling, using qRT-PCR [57] or small RNA sequencing (NGS) [58]. In both profiling studies, results were validated by qRT-PCR for each single miRNA. While Benigni and colleagues found eight out of fourteen miRNAs as significantly deregulated, Waller and coworkers failed to confirm statistically significant differences in miRNA expression [57,58].

A common feature observed by the authors is an overall down-regulation of miRNAs in CSF samples from ALS patients [55,57,58], in agreement with other studies showing that the majority of deregulated miRNAs in tissues from ALS models and ALS patients are down-regulated [59]. This could suggest a general default in RNA metabolism in ALS [38].

In general, however, these studies highlight a wide heterogeneity among miRNAs significantly deregulated. A possible explanation could be the variability in terms of experimental approach and technical procedures and the reduced number of CSF samples analyzed in each study.

Some authors evaluated the correlation between CSF and serum miRNA expression levels. A significant positive correlation between expression levels in CSF and serum from ALS patients was found for miR-338-3p [56] and miR-143-3p [55]. However, the amount of most miRNAs was independently regulated between the two biofluids at individual level. This suggests that CSF miRNAs do not simply reflect the usually more abundant serum miRNAs, and changes in the serum do not necessarily reproduce alterations of CSF levels [55].

It should be noted that, among the different body fluids, the lowest abundance of miRNAs appears in CSF. Thus, it is possible that some potentially promising and informative miRNAs, identified both in vivo and in vitro ALS models, are below the limit of detection of the available methods of analysis. For example, the miR-218, a motor neurons-enriched miRNA, has been found increased in CSF of ALS rodent models: its expression correlates with the number of remaining spinal motor neurons and is responsive to motor neuron sparing therapy [60]. miR-218 could thus represent a potential biomarker to assess drug effects on motor neurons during clinical trials in ALS patients. However, at the present time, this miRNA is detectable only in some CSF samples, and thus a comparison between ALS patients and controls is not possible.

An approach to overcome the technical limits due to low abundance of several miRNAs in CSF could be to focus on those miRNAs found up-regulated in ALS patients. Among these, miR-338-3p seems to be very promising, since it has been reported as consistently upregulated in CSF, serum and leukocytes from ALS patients [56]. miR-338-3p is involved in several molecular pathways and could contribute to ALS pathogenesis through different modalities, such as neurodegeneration and apoptosis. Recent evidence suggests that miR-338 participates in the control of neuroblast apoptosis and in neuroblastoma pathogenesis [61] and it is able to suppress neuroblastoma proliferation, invasion and migration [62]. Interestingly, also another miRNA found up-regulated in CSF from ALS patients, miR181a-5p, has been proposed as an anti-oncomir, which acts as a tumor suppressor in normal tissues, promoting growth inhibition and apoptosis [63]. These findings suggest that these up-regulated miRNAs are involved in ALS pathogenetic process through apoptotic mechanisms responsible for cell death.

In order to increase diagnostic accuracy, up-regulated miRNAs can be used in combination with other miRNAs, identified as down-regulated. Benigni and colleagues reported that the ratios of miR-181a-5p/miR-15b-5p and miR-181a-5p/miR-21-5p considerably increased the specificity with a slight decrease in sensitivity compared with each individual miRNA [57]. A wider use of this strategy could allow improvements in the performance of identified biomarkers and should be taken into account for future studies, as further discussed in the following paragraphs.

### 4.2. Circulating miRNAs

The use of blood samples in the diagnostic routine presents several advantages. Blood specimens are easy to obtain, process and store, and the samples required for the analysis can be collected without using invasive procedures for the patients. The lack of ethical implications as compared with CSF and muscle biopsy makes it possible to repeat the blood draw during the disease progression. Since miRNAs circulate in the blood in a highly stable form, this may facilitate the procedure of storage and conservation and increase the flexibility of the analysis.

Blood-based biomarkers may originate from the CNS through a transfer between the blood and CSF at the blood–CSF barrier [64,65], suggesting that the same biomarkers could be present in both biofluids. They may be generated also by other organs and tissues affected during ALS, such as degenerating muscles or peripheral blood cells. Therefore, blood can represent an excellent biofluid for discovery and validation of biomarkers for ALS [66]. On the other hand, miRNAs present in blood can reflect other pathophysiological conditions concurrent but not directly related to ALS disease (e.g. inflammatory status, response to pharmacological treatments, etc.), which may represent confounding factors.

Several studies on circulating miRNAs as potential biomarkers for ALS have been published. The findings from such studies are summarized in Table 2.

As reported in Table 2, several studies have identified numerous potential miRNA biomarkers in peripheral blood from ALS patients, however their results rarely overlap with each other. This high discrepancy in the identified miRNAs is probably associated with the variability of quantification methods, miRNA normalizers used, number of samples included, clinical features of patients, and also with the differences in selected source of miRNAs (serum, plasma, leukocytes, and whole blood). Another possible reason for the poor reproducibility of results may be the high level of heterogeneity in miRNA profiles of SALS patients in comparison to FALS patients. Freischmidt and colleagues initially reported a signature of 22 miRNAs significantly down-regulated in FALS and presymptomatic mutation carriers [68]. Subsequently, the same authors replicated the analysis of these miRNAs in a larger SALS sample group using identical technical procedures, and found only 2 miRNAs significantly down-regulated in all SALS patients. A more accurate analysis of results revealed that around 60% of SALS patients shared a serum miRNA fingerprint with genetic cases, while the remaining around 40% of patients were evenly distributed among control samples. The absence of FALS-like miRNA patterns in these patients may mirror a higher impact of exogenous factors and possibly a lower and/or different genetic influence in a subgroup of SALS patients [69].

Interestingly, the miRNA expression profiles derived from the study performed by Freischmidt and colleagues [68] were re-elaborated applying principal component analysis (PCA)-based unsupervised feature extraction (FE), another analysis approach [81]. The authors identified a total of 51 deregulated miRNAs, 27 down-regulated and 24 up-regulated in ALS patients in comparison with healthy controls. Applying the linear discriminant analysis (LDA) to these selected miRNAs, overall accuracy was 0.66 including healthy controls, ALS mutation carriers, FALS and SALS patients. Of note, excluding SALS patients, LDA was able to successfully discriminate healthy controls, ALS mutation carriers and FALS patients, with an accuracy rising up to 0.84, confirming as the heterogeneity of SALS group can introduce a wider variability in circulating miRNA profiles.

Among the studies published until now, a largely used approach is miRNA profiling on blood samples from ALS patients and controls, carried out by microarray [51,68,69,70,71], PCR-array [75,76] and NGS [80]. Other studies performed analysis on specific miRNAs, selected from data previously reported in the literature [55,56,73,74,77]. In other cases, the first step of the research was a microarray analysis on samples from transgenic mice [54,67,78] or skeletal muscle biopsies from ALS patients [68], followed by validation of miRNAs found deregulated in the first step of analysis.

Only one study analyzed miRNA expression specifically in serum exosomes [79]. Exosomes are double lipid vesicles secreted by a variety of cells and widespread in the peripheral body fluid. They can reflect physiological and pathological changes of the cells of origin, representing potential new biomarkers for disease diagnosis [82]. miRNAs are enriched in exosomes, and the exosome membrane structure can protect them from degradation by RNA enzymes. The authors investigated the expression of only miR-27a-3p, previously reported as present in myoblast-derived exosomes [83], and found a down-regulation of this miRNA in ALS patients, suggesting that miRNA exosome analysis could represent a future perspective for ALS biomarker identification.

Despite a poor overlapping among the miRNAs identified as deregulated in ALS, some circulating miRNAs seem to be particularly promising as potential biomarkers in ALS patients. Table 3 summarizes these miRNAs, reported as de-regulated in two or more papers.

As shown in the Table 3, some common pathways emerge: some miRNAs are involved in neurodegeneration and apoptosis (miR-338, miR-142, miR-183 and let-7d), other miRNAs act at muscle level (miR-206, miR-133a, miR-133b and miR-27a). In particular, miR-206, miR-133a and miR-133b are myo-miRNAs, molecules specifically expressed in striated muscle and involved in muscle proliferation, repair and regeneration. Their expression levels change during the process of myogenesis, development, atrophy, degeneration, and myopathies [84]. The more recurrent result is an up-regulation of circulating miR-206 in ALS patients. miR-206 is a human skeletal muscle-specific miRNA that promotes the formation of new neuromuscular junctions following nerve injury, and therefore plays a crucial role in the reinnervation process [85]. In miR-206 knock-out mice, delayed and incomplete muscular reinnervation was observed in comparison to those animals that expressed miR-206. In addition, high expression levels of miR-206 were found in a mouse model of ALS, and its under-expression was associated with a faster progression of the disease [86]. A consensus for higher expression levels of this miRNA in ALS patients compared to controls was reported by several authors [67,72,73,74,76,77]. Although miR-206 seems to represent a valid circulating biomarker for ALS, it is still to define whether the elevated expression of this miRNA is the result of the disease or its cause.

While all the works performed a comparison between samples from ALS patients and healthy controls, only a subset of them included also samples from patients affected by other neurological disorders. Neurological controls comprised Parkinson’s disease [56,70,71,74], Alzeihmer’s Disease [56,69,74,75], Huntington’s disease [56,69,71], Multiple Sclerosis [54,75] and ALS-mimic conditions [76]. The use of neurological controls can help to discriminate whether identified miRNAs are really specific for ALS or are common features linked to neurodegenerative processes. For example, the comparison of miRNA expression between ALS and Parkinson’s disease patients suggested that miR-183 might be specific for SALS, whereas miR-451 and miR-3935 might be more general biomarkers linked to neurodegenerative disorders [71]. In addition, the inclusion of an ALS-mimic patients’ group may contribute to identify miRNA biomarkers to use in the differential diagnosis in the early stages of the disease.

Only a part of the studies performed until now investigated the potential correlations among miRNA expression levels and ALS clinical features, sometimes in longitudinal studies, measuring miRNA levels in the same ALS patient over time [70,72,75,76,80]. In some case this analysis failed to find any association [67,69,77], in other cases specific correlations were reported. Some authors described associations of miRNA expression levels with ALS site of onset [70,74,76,80], ALS Functional Rating Scale-revised (ALSFRS-R) and/or vital capacity (VC) [70,75,78,80], Medical Research Council (MRC) sumscore [72] and with the disease progression rate [72,80]. Only two studies investigated the possible associations of specific serum miRNAs with riluzole treatment, failing to identify any correlation [75,76]. Such results must be anyway considered with caution, since the number of subjects included in every group is limited. They need to be confirmed in larger cohorts of ALS patients, to really define the role of miRNA expression in ALS clinical presentation and progression. From this perspective, it would be very important that, after the identification of potential miRNA biomarkers, more longitudinal studies were performed, to evaluate if these miRNAs could be used as prognostic indicators.

As already mentioned for CSF studies, also in serum the analysis of combinations of several miRNAs has shown a higher accuracy than single miRNAs in discriminating ALS from healthy controls or other neurological disorders [71,74,75]. A very interesting approach is reported by Sheinerman and colleagues, who developed a strategy based on miRNA pairs, consisting of one miRNA enriched in synapses of a brain region affected by the disease and another miRNA enriched in a different brain region or cell type. The use of the pair of miRNA derived from the same organ allowed decreasing potential overlap with pathologies of other organs and reducing also inter-individual variability. The authors demonstrated that, combining two or three effective miRNA pairs into a single miRNA classifier, they could achieve a greater accuracy in discriminating ALS both from healthy controls and patients affected by other neurological disorders [74]. Thus, in the future studies it should be considered that, while the deregulation of a single miRNA can be a feature common to several neurological diseases, panels of deregulated miRNAs, or combinations of them, may result highly specific for ALS and represent a signature for this disease.

Finally, a relevant aspect of the use of miRNAs as ALS biomarkers is their potential of identifying the disease in very early stages, also before any clinical manifestation. In their work, Freischmidt and colleagues showed that a specific subset of miRNAs, reduced in the serum of patients with familial and sporadic ALS, was reduced also in presymptomatic carriers of pathogenic ALS mutations. Moreover, the down-regulation was largely independent of the underlying disease gene and was stronger in patients with familial ALS than in pre-manifest mutation carriers, suggesting that alterations of miRNA profiles could be progressive when comparing the pre-manifest and manifest phase of the disease [68]. If confirmed, these findings may be of fundamental importance for the development of screening tests able to detect ALS in early asymptomatic stages and for future preventive therapeutic strategies before the occurrence of clinically evaluable symptoms.

### 4.3. miRNAs in Muscle Biopsies

Skeletal muscle is another potential source for the identification of candidate miRNA biomarkers. In the last years, it has become evident that ALS does not affect only motor neurons but also other cell types, including striated muscle, which play an active role in the disease pathogenesis. Before the clinical onset and during the disease progression, the affected skeletal muscle of ALS patients attempts to restore function by futile cycles of reinnervation and denervation [87]. Eventually, persistent muscle wasting exceeds the ability to repair and consequently the atrophy process starts. Due to the crucial role of the skeletal muscle in ALS pathogenesis, recent studies have focused their research on the identification of specific muscle miRNAs in ALS tissues, which could potentially be use as prognostic biomarkers of disease. Moreover, miRNAs identified in skeletal muscle of ALS patients could be used as biomarkers also in plasma or serum, where they can be released by the affected tissues. This strategy seems to be particularly interesting, since muscle biopsy is unfortunately an invasive practice and cannot be proposed for longitudinal studies to follow disease progression.

Several studies focused on analysis of myo-miRNAs, including miR-1, miR-133a, miR-133b, miR-206, miR-208a, miR-208b, miR-499, and miR-486 [88]. Most of them explored the role of these miRNAs in mouse models (for a review see [89]), but only few studies investigated the role of these molecules as possible markers in muscle biopsies of patients with ALS, due to the rarity and difficulty to obtain this kind of samples. miRNAs found deregulated in muscle biopsies from ALS patients compared to healthy control subjects are shown in Table 4. 

Most studies focused on the expression of myo-miRNAs [90,91,92,93]; only in some cases also other miRNAs were included, for example miRNAs related to inflammation/angiogenesis [93] or selected by microarray [72] or NGS approaches [94,95]. Overall, results are sometimes contrasting and poorly reproducible. These non-concordant finding could be attributed to different types of muscle used for biopsy, discordance among the samples in terms of inclusion criteria of patients (age, gender, evolution of disease, onset) and different techniques and internal control molecules used to assess miRNA expression levels.

Some authors performed also a correlation analysis among miRNA expression and ALS clinical features. Table 5 reports miRNAs altered in tissue of specific stratified ALS patients’ groups analyzed in comparison to control subjects.

In addition, in other papers the associations with clinical variables were analyzed comparing groups of patients to each other. Stratifying ALS patients, an up-regulation of myo-miRNAs (miR -206, miR-133a, miR-133b and miR-27a) and of inflammatory miRNAs (miR-155, miR-146a and miR -221) was discovered in ALS patients with earlier age at onset (<55 years) and longer disease duration [93]. Moreover, significantly higher expression levels of the same myo-miRNAs and inflammatory miRNAs were detected in male than in female. This gender difference has been hypothesized to be related to a difference in hormonal regulation, implying a slower disease progression in women [93]. In another paper, miR-29c, miR-208b and miR-499 were reported as increased in patients with slow disease course [96]. Expression data were analyzed in patients categorized into “early” and “late” based on disease duration at the moment of biopsy (more or less one year). miR-9 and miR-206 significantly increased in the early patients’ group and, of note, miR-206 inversely correlated with the time from symptoms onset to muscle biopsy, indicating an early response to denervation in skeletal muscle [96].

Although the results are often inconsistent among different studies, some trends in miRNAs deregulation seem to emerge. One of the most interesting miRNA is miR-133a, which was found to be up-regulated in human ALS tissues [93,95,96], particularly in patients with slow disease progression and in biopsies obtained before one year from the symptom onset [96]. At the same time, a significant reduction of this miRNA was present in a specific ALS patients’ group with higher disease severity [95], suggesting changes in its expression during the disease progression. In contrast, however, other studies detected a down-regulation of miR-133a in human biopsies, as reported also in mice [92,94]. At the moment, the strongest data are those concerning miR-206. Indeed, the mechanisms responsible for the increase of this miRNA seem to be conserved in the skeletal muscle of mouse models and in that from ALS patients, and the up-regulation described in both cases is an ALS-specific response to the denervation. miR-206 was found significantly up-regulated in muscle samples from ALS patients compared to control subjects [72,90,91,93], similarly to what observed in blood samples, strengthening the role of this miRNA as potential biomarker for ALS. Of note, miR-206 showed an increased trend in muscle biopsies from long-term survivor patients, even though below the statistically significance [90]. De Andrade and colleagues [72] reported that this miRNA was over-expressed both in plasma and skeletal muscle of patients with ALS, but the over-expression was not progressive during the follow-up. They supposed that miR-206 expression increased early in the disease course, reaches a plateau and then begins to fall. In agreement with this hypothesis, an up-regulation of miR-206 was described in muscle biopsies from ALS patients within one year from the clinical onset, becoming less evident as the disease progresses to a later stage [96]. Finally, Si and collaborators reported a non-significant upward trend in miR-206 in muscle samples from ALS patients compared to controls. This result, however, was correlated to a high standard error for this miRNA due to the variability among samples. Moreover, the authors reported a significant inverse correlation between this miRNA and the muscle power of the biopsied muscle, hypothesizing that it could be a marker of disease activity. This finding highlights the importance to associate miR-206 levels with muscle-specific clinical assessment rather than overall clinical status [94].

Although a concordant miRNA signature have not been identified yet in ALS patient muscle biopsies, these findings show that miRNAs could be useful prognostic markers to better understand the course of disease. In particular, the identification of a specific muscular miRNA profile through multicenter studies, able to increase the statistical power of the analysis, could lead to a stratification of the patients in order to identify prognostic biomarkers to use as indicator of disease progression, facilitating the clinical management of patients.

## 5. Conclusions and Future Perspectives

Despite the intense research activity of the last years, the use of miRNAs as biomarkers for diagnosis of ALS and clinical management of patients is still in an early stage of development. Several interesting data have been obtained so far, with important insights into the disease processes. However, results achieved in different studies are most of the time conflicting and poorly reproducible, making it difficult to unequivocally identify which miRNA(s) may be selected as biomarker in clinical practice. In order to overcome these limits, some improvements in the research approach should be taken into account.

First of all, one factor strongly complicating the comparison among data reported by different research groups is the wide range of methods used for the identification of potential miRNA biomarkers and the different techniques for miRNA measurement and data normalization. A common acceptance of certain guidelines, standard research protocols, and strong methods of statistical analysis of miRNAs will be important in the future to achieve reliable biomarkers.

Another critical issue is the relatively small number of patients included in the studies performed until now. Results are often interesting, but they need to be verified in larger cohorts of ALS patients. It would be really important that those miRNAs, which have shown initial promise, were validated in independent laboratories and/or in multicenter collaborations. In addition, since ALS is a highly heterogeneous disease, replication studies should increase the number of patients stratifying them based on clinical and genetic features, in order to obtain a better assessment of the potential associations among miRNAs and these variables.

Further, in several studies miRNA levels of ALS patients have been compared only to those of control subjects not affected by neurological disorders. This approach may bring to the identification of miRNAs able to successfully differentiate patients from healthy control subjects, but these miRNAs are often associated with common pathologic processes of neurodegeneration and are not specific for ALS. It will be of fundamental importance to extend the comparison to patients affected by other neurodegenerative diseases, in particular ALS-mimic disorders, to evaluate the specificity of deregulated miRNAs for ALS.

One of the more interesting approaches to miRNA biomarker identification is the use of a complex set of biomarkers, or combinations or ratios of biomarkers from different pathogenic pathways, rather than the employ of a single marker. This strategy has been shown to increase the sensitivity and/or specificity of potential ALS biomarkers and to contain more exhaustive diagnostic information, and should be more widely used in future researches.

At the same time, when possible, future studies should try to combine data obtained from multiple source of sample (blood, CSF, muscle) of the same patient. Up to date, only few studies have performed this kind of analysis, and their results are quite conflicting. However, an extensive analysis of correlations among different samples could be helpful to obtain more informative data and improve patients’ stratification. In addition, for circulating miRNAs, it would be important to perform longitudinal studies on a large number of patients, in order to identify potential biomarkers of disease progression, and evaluate their role as prognostic indicators.

In conclusion, miRNAs constitute very promising biomarkers for ALS, but there is still much work to be done to validate and use them in clinical routine. The ultimate objective is to include these biomarkers in all phases of ALS management, from the diagnosis to the clinical trials, and, in perspective, to the identification of future therapeutic approaches.

## Figures and Tables

**Table 1:** Deregulated microRNAs (miRNAs) in cerebrospinal fluid (CSF) of amyotrophic lateral sclerosis (ALS) patients compared to healthy controls.

miRNA (Hsa-miR)	miRNA Expression Change	No. of Specimens	miRNA Detection Approach	Ref.
150, 99b, 146a 27b, 328, 532-3p	↑ in SALS ↑ in SALS and FALS	SALS: 10 FALS: 5 HCs: 10	qRT-PCR	[54]
132-5p, 132-3p, 143-3p 143-5p, 574-5p	↓ ↑	SALS: 22 HCs: 24	qRT-PCR	[55]
338-3p	↑	SALS: 10 HCs: 10	qRT-PCR	[56]
181a-5p 21-5p, 195-5p, 148-3p, 15b-5p, let7a-5p, let7b-5p, let7f-5p	↑ ↓	SALS: 24 HCs: 24	qRT-PCR	[57]
124-3p, 127-3p, 143-3p, 125b-2-3p, 9-5p, 27b-3p 486-5p, let7f-5p, 16-5p, 28-3p, 146a-3p, 150-5p, 378a-3p, 142-5p, 92a-5p	↑ ↓	SALS: 32 HCs: 10 NCs: 6	NGS	[58]

Abbreviations: Ref., Reference; ↑/↓, up-regulated/down-regulated; SALS, sporadic amyotrophic lateral sclerosis patients; FALS, familial amyotrophic lateral sclerosis patients; HCs, healthy controls; NCs, neurological disease control subjects (multiple sclerosis); qRT-PCR, quantitative Real-Time Polymerase Chain Reaction; NGS, Next Generation Sequencing.

**Table 2:** Deregulated circulating microRNAs (miRNAs) in amyotrophic lateral sclerosis (ALS) patients compared to healthy controls.

miRNA (Has-miR) Expression Change	Source	miRNA Detection Approach	No. of Specimens for miRNAs Validation	Ref.
↑: 338-3p	Leukocytes	Microarray→ miRNAs validation with qRT-PCR	SALS: 14 HCs: 14	[51]
↑: 27a, 155, 142-5p, 223, 30b, 532-3p	Monocytes (CD14+ CD16-)	Nanostring nCounter ^1^ → miRNAs validation with qRT-PCR	SALS: 22 FALS: 4 HCs: 24	[54]
↓: 132-3p, 132-5p, 143-3p, 143-5p, let-7b	Serum	Nine *TARDBP* binding miRNAs and miR-9-5p → qRT-PCR	SALS: 22 HCs: 24	[55]
↑: 206, 106b	Serum	Microarray ^1^ → miRNAs validation with qRT-PCR	SALS: 12 HCs: 12	[67]
↑: 338-3p	Leukocytes and serum	miR-338-3p → qRT-PCR	SALS: 10 HCs: 10	[56]
↓ in FALS/SALS: 4745-5p, 3665, 4530 ↓ in FALS: 1915-3p	Serum	Microarray → miRNAs validation with qRT-PCR	FALS: 23 HCs: 24 SALS: 14 HCs: 14	[68]
↓ in FALS/SALS: 1825 ↓ in SALS: 1234-3p	Serum	Microarray→ miRNAs validation with qRT-PCR	SALS: 20 HCs: 20 FALS: 13 HCs: 13	[69]
↑: 4649-5p ↓: 4299	Plasma	Microarray → miRNAs validation with qRT-PCR	SALS: 48 HCs: 47	[70]
↓: 183, 193b, 451, 3935	Leukocytes	Microarray → miRNAs validation with qRT-PCR	SALS: 83 HCs: 61	[71]
↑: 424, 206	Plasma	Microarray ^2^ → miRNAs validation with qRT-PCR	SALS: 39 HCs: 39	[72]
↑: 206, 133a,133b ↓: 146a, 149*, 27a	Serum	Preselected myo-miRNAs, inflammatory and angiogenic miRNA → qRT-PCR	SALS: 14 HCs: 8	[73]
↑: 206 Deregulated MicroRN pairs: 206/338-3p 9*/129-3p 335-5p/338-3p	Plasma	Thirty seven brain-enriched and inflammation-associated microRNAs → qRT-PCR	ALS: 50 HCs: 50	[74]
↑ ^┼^: 1, 133a-3p, 133b, 144-5p, 192-3p, 195-5p, 19a-3p ↓ ^┼^: let-7d-3p, 320a, 320b, 320c, 425-5p, 139-5p	Serum	qRT-PCR array	SALS: 20 FALS: 3 HCs: 30 NCs: 103	[75]
↑: 206, 143-3p ↓: 374b-5p	Serum	qRT-PCR array → miRNAs validation with qRT-PCR	SALS: 23 CRL: 22	[76]
↑: 9, 338, 638, 663a, 124a, 451a, 132, 206, let-7b	Leukocytes	Preselected 10 miRNAs → miRNAs validation with qRT-PCR	SALS: 84 HCs: 27	[77]
↑: 142-3p ↓: 1249-3p	Serum	Microarray ^1^ → miRNAs validation with qRT-PCR	SALS: 20 HCs: 20	[78]
↓: 27a-3p	Serum exosomes	miR-27a-3p → qRT-PCR	ALS: 10 HCs: 20	[79]
↓: let-7a-5p, let-7d-5p, let-7f-5p, let-7g-5p, let-7i-5p, 103a-3p, 106b-3p, 128-3p, 130a-3p, 130b-3p, 144-5p, 148a-3p, 148b-3p, 15a-5p, 15b-5p, 151a-5p, 151b, 16-5p, 182-5p, 183-5p, 186-5p, 22-3p, 221-3p, 223-3p, 23a-3p, 26a-5p, 26b-5p, 27b-3p, 28-3p, 30b-5p, 30c-5p, 342-3p, 425-5p, 451a, 532-5p, 550a-3p, 584-5p, 93-5p	Whole blood	NGS → qRT-PCR	SALS: 50 HCs: 15	[80]

**^1^** analysis carried out on samples from transgenic mice; ^2^ analysis carried out on samples of ALS patients’ skeletal muscle biopsies; ^┼^, miRNAs deregulated in ALS patients compared to healthy controls and neurological controls (including multiple sclerosis and Alzheimer’s disease patients). Abbreviations: Ref., Reference; ↑/↓, up-regulated/down-regulated; SALS, sporadic amyotrophic lateral sclerosis patients; FALS, familial amyotrophic lateral sclerosis patients; HCs, healthy controls; NCs, neurological controls; qRT-PCR, quantitative Real-Time Polymerase Chain Reaction; NGS, Next Generation Sequencing.

**Table 3:** The most promising circulating microRNAs (miRNAs) detected as potential biomarkers in amyotrophic lateral sclerosis (ALS) patients.

miRNAs (Has-miR)	miRNA Change	Role in ALS	Ref.
206	↑	Myo-miRNA: muscle proliferation, repair and regeneration. It promotes neuromuscular connectivity and enhances reinnervation	[67,72,73,74,76,77]
338	↑	Involvement in different pathways such as apoptosis, neurodegeneration, and/or glutamate clearance	[51,67,74,77]
133a	↑	Myo-miRNA: muscle proliferation, repair and regeneration	[73,75]
133b	↑	Myo-miRNA: muscle proliferation, repair and regeneration	[73,75]
142	↑	miRNA predicted to target a specific set of genes associated to the pathophysiology of ALS, including *TARDBP* and *C9orf72*.	[54,78]
183	↓	miRNA involved in neurodegenerative signaling pathway, including PI3K-Akt and MAPK pathway. miR-183/mTOR pathway contributes to spinal muscular atrophy pathology	[71,80]
27a	↓	miRNA involved in muscle growth, myoblast proliferation acting on myostatin. It is present in myoblast-derived exosomes	[73,79]
let-7d	↓	Involvement in apoptosis by the Hippo signaling pathway	[75,80]

Abbreviations: Ref., Reference; ↑/↓, up-regulated/down-regulated.

**Table 4:** Deregulated miRNAs in skeletal muscle biopsies of amyotrophic lateral sclerosis (ALS) patients compared to healthy controls.

miRNA (Hsa-miR)	miRNA Expression Change	Type of Muscle	No. of Muscle Biopsies	miRNA Detection Approach	Ref.
206	↑	Deltoid, anconeus	FALS: 1 SALS: 10 HCs: 6	mir-206 → qRT-PCR	[90]
23a, 29b, 206, 455, 31	↑	Vastus lateralis	ALS: 14 HCs: 10	Myo-miRNAs and miRNAs dysregulated in human muscle disease → qRT-PCR	[91]
1, 26a, 133a, 455	↓	Vastus lateralis	ALS: 5 HCs: 7	Myo-miRNAs → qRT-PCR	[92]
424, 214, 206	↑	Biceps brachii	ALS: 5 HCs: 5	Microarray → miRNAs validation with qRT-PCR	[72]
1, 206, 133a, 133b, 27a, 155, 146a, 221	↑	Quadriceps femoris	SALS: 13 HCs: 5	Inflammatory/angiogenic miRNAs and myo-miRNAs → qRT-PCR	[93]
1, 10b-5p, 100-5p, 133a-3p, 133b-3p	↓	Biceps, deltoid, tibialis anterior, vastus lateralis	ALS: 19 HCs: 9	NGS ^1^ and qRT-PCR ^1^ → qRT-PCR	[94]
100-5p, 10a, 125a-5p, 133a-1/-2-3p, 362, 500a-3p, 542-5p, 99a-5p 1303-3p, 150-5p, 26a-1/-2-5p, 486-1/-2-5p,	↑ ↓	Vastus lateralis	FALS: 2 SALS: 9 HCs: 11	NGS	[95]

**^1^** analysis carried out on samples from transgenic mice. Abbreviations: Ref., Reference; ↑/↓, up-regulated/down-regulated; SALS, sporadic amyotrophic lateral sclerosis patients; FALS, familial amyotrophic lateral sclerosis patients; HCs, healthy controls; qRT-PCR, quantitative Real-Time Polymerase Chain Reaction; NGS, Next Generation Sequencing.

**Table 5:** Deregulated miRNAs in skeletal muscle biopsies of specific amyotrophic lateral sclerosis (ALS) patients’ groups analyzed in comparison to healthy controls.

miRNA (Hsa-miR)	miRNA Expression Change in Specific ALS Patients’ Group	Type of Muscle	No. of Muscle Biopsies	miRNA Detection Approach	Ref.
133a, 29c, 9, 208b 1, 208b 133a, 133b, 206, 29c, 9, 155, 23a	↑in ALS slow group ^1^ ↓ in ALS rapid group ^2^ ↑ in early stage group ^3^	Deltoid and quadriceps	FALS: 3 SALS: 11 HCs: 24 Slow group ^1^: 6 Rapid group ^2^: 5 Early group ^3^: 4 Late group ^4^:9	Eleven skeletal muscle related miRNAs → qRT-PCR	[96]
100-5p, 199a-1/-2, 199b-3p, 27a-5p, 3607-3p, 424-5p, 450a-1/-2-5p, 450b-5p, 501-3p, 502-3p, 542-5p, 660-5p 1303-3p, 133a-1/-2-3p, 150-5p, 378, 486-1/-2-5p, 502-3p, 855-3p	↑ in higher disease severity ^5^ ↓ in higher disease severity ^5^	Vastus lateralis	Higher disease group ^5^: 7 HCs: 11	NGS	[95]

**^1^**, ALS slow group (≥4 years of disease progression without requiring respiratory supports); ^2^, ALS rapid group (<4 years of disease progression without respiratory supports or death occurring <4 years from symptoms onset); ^3^, early stage group (less than one year from symptom onset to muscle biopsy); ^4^, late stage group (more than one year from symptom onset to muscle biopsy); ^5^, group of patients with higher disease severity. Abbreviations: Ref., Reference; ↑/↓, up-regulated/down-regulated; SALS, sporadic amyotrophic lateral sclerosis patients; FALS, familial amyotrophic lateral sclerosis patients; HCs, healthy controls; qRT-PCR, quantitative Real-Time Polymerase Chain Reaction; NGS, Next Generation Sequencing.
